# LncRNA MIR4435-2HG-mediated succinylation of USF1 promotes its protein stability and induces epithelial-mesenchymal transition in HNSCC

**DOI:** 10.1080/15592294.2026.2672218

**Published:** 2026-05-14

**Authors:** Shujin He, Jing Wang, Dingding Wang, Wei Wang, Anjia Han, Honggang Liu

**Affiliations:** a Department of Pathology, the First Affiliated Hospital, Sun Yat-Sen University, Guangzhou, China; b Department of Pathology, Beijing Tongren Hospital, Capital Medical University, Beijing, China; c Department of Pathology, National Cancer Center/National Clinical Research Center for Cancer/Cancer Hospital, Chinese Academy of Medical Sciences and Peking Union Medical College, Beijing, China; d Department of Neonatal Surgery, Beijing Children’s Hospital, Capital Medical University, Beijing, China; e Department of Pathology, Affiliated Hospital of Jining Medical University, Jining Medical University, Jining, Shandong, China

**Keywords:** Head and neck squamous cell carcinoma, metastasis; lncRNA MIR4435-2HG, USF1, succinylation

## Abstract

Tumor recurrence and metastasis remain the principal causes of mortality in patients with head and neck squamous cell carcinoma (HNSCC). However, the key molecular drivers underlying HNSCC progression and their regulatory mechanisms have not yet been fully elucidated. In this study, we investigated the biological role of the long noncoding RNA MIR4435-2HG in HNSCC. Integrated analyses across multiple public databases demonstrated that MIR4435-2HG is significantly upregulated in HNSCC and is closely associated with pathological T stage, clinical stage, and histological grade. Through further bioinformatic screening and functional validation, upstream transcription factor 1 (USF1) was identified as a critical downstream effector of MIR4435-2HG. Mechanistically, MIR4435-2HG directly interacts with USF1 and promotes its protein stability by facilitating USF1 succinylation, thereby enhancing tumor cell migration and invasion through the induction of epithelial – mesenchymal transition. Moreover, dual-luciferase reporter assays and RT-qPCR analyses revealed that CREB1 transcriptionally upregulates MIR4435-2HG expression, establishing a positive feedback regulatory circuit. Collectively, our findings demonstrate that MIR4435-2HG promotes USF1 succinylation and stabilization, leading to the formation of a MIR4435-2HG – USF1–AKT – CREB1 signalling loop that drives epithelial – mesenchymal transition and facilitates the invasive and metastatic potential of HNSCC cells.

## Introduction

Head and neck squamous cell carcinoma (HNSCC) is one of the sixth most common cancers in the world, with more than 850,000 cases and 400,000 deaths per year [1]. Originating from the mucosal squamous epithelium of the upper aerodigestive tract, HNSCC most frequently involves the oral cavity, nasal sinuses, oropharynx, hypopharynx, larynx, and tonsils [2,3]. Although multimodal treatment strategies combining surgery, radiotherapy, and chemotherapy have improved locoregional disease control, the long-term outcomes of patients with locally advanced HNSCC remain suboptimal. Tumor recurrence and distant metastasis occur in 40–50% of cases and represent the primary causes of disease-related mortality [4,5]. These clinical challenges underscore an urgent need to identify molecular biomarkers and regulatory pathways that not only improve risk stratification but also provide actionable therapeutic targets.

Long noncoding RNAs (lncRNAs) have emerged as critical regulators of gene expression and cellular signalling in cancer. Despite their generally low expression abundance, lncRNAs display high tissue and disease specificity, rendering them particularly attractive as biomarkers and potential therapeutic targets [6,7]. Increasing evidence indicates that dysregulated lncRNAs contribute to key oncogenic processes, including epithelial – mesenchymal transition (EMT), metabolic reprogramming, and resistance to therapy, thereby directly influencing tumor aggressiveness and clinical outcome. Among these, the lncRNA MIR4435-2HG was initially identified by Jiang and colleagues in 2015 as a transcript highly expressed in lung cancer and involved in the antitumor effects of resveratrol [8]. Subsequent studies have demonstrated aberrant upregulation of MIR4435-2HG across multiple malignancies, as well as in patient-derived peripheral blood samples, suggesting its potential utility as a minimally invasive biomarker. Functionally, MIR4435-2HG has been implicated in tumor proliferation, invasion, and metastasis through diverse molecular mechanisms, although these appear to be context dependent. Importantly, the expression pattern, upstream regulation, and mechanistic role of MIR4435-2HG in HNSCC have not yet been systematically investigated.

Recent advances in RNA-based therapeutics have highlighted the potential of targeting noncoding RNAs in cancer. Approaches such as small interfering RNAs (siRNAs), antisense oligonucleotides (ASOs), and microRNA-based strategies enable selective modulation of oncogenic RNA networks and have shown promising translational potential [9–11]. Given the high tissue specificity and regulatory versatility of lncRNAs, these molecules are increasingly being explored as therapeutic targets in precision oncology. However, whether MIR4435-2HG represents a viable target for RNA-based intervention in HNSCC remains unclear.

Given the central role of transcriptional regulation and post-translational modification in cancer progression, we hypothesized that MIR4435-2HG may exert its oncogenic effects in HNSCC by modulating the stability and activity of key transcription factors involved in tumor invasion and metastasis. In the present study, we aimed to delineate the regulatory network controlling MIR4435-2HG expression and to elucidate its mechanistic contribution to HNSCC progression.

## Materials and methods

### Patient datasets

The expression data as well as the patient clinical information, which included 502 HNSCC samples, 44 nontumor samples and 43 paired samples of HNSCC patients were downloaded from TCGA (www.tcga-data.nci.nih.gov/tcga/) using R package. In addition, five fresh tumor tissues as well as paired adjacent normal tissues of laryngeal squamous cell carcinoma (LSCC) patients were collected from Beijing Tongren Hospital, Capital Medical University (Beijing, China).A total of 75 formalin-fixed, paraffin-embedded tumor specimens from patients with HNSCC were obtained from the Affiliated Hospital of Jining Medical University. Beijing Tongren Hospital and the Affiliated Hospital of Jining Medical University granted Ethical approval to carry out the study within its facilities (version number 20180212 and 2021C089). This study was conducted in accordance with the principles of the Declaration of Helsinki. The requirement for informed consent was waived due to the retrospective nature of the study.

### Cell culture

Human laryngeal squamous cell carcinoma cells TU212 and TU686 were originally sourced from the American Type Culture Collection (ATCC) in Mar. 2019, and were separately authenticated using short tandem repeat (STR) in Feb. 2021 and Oct. 2019. In addition, human laryngeal squamous cell carcinoma cells SNU899 and SNU1076 were sourced from Korea Cell Line Bank in May 2022, and were passaged no more than ten times for use in experiments. SNU899 and SNU1076 were cultured in Iscove’s Modified Dulbecco’s Medium (KeyGEN BioTECH, KGM12200N-500) supplemented with 10% fetal bovine serum (BI, #04–001-1ACS). TU212 were cultured in Iscove’s Modified Dulbecco’s Medium (KeyGEN BioTECH, KGM12200N-500) supplemented with 10% fetal bovine serum (Ausbin, WS500T). Human laryngeal squamous cell carcinoma cells TU686 was cultured in RPMI 1640 with Glutamine (HyClone, SH30809) supplemented with 10% fetal bovine serum (BI, #04–001-1ACS). Human pharynx squamous cell carcinoma cell line FaDu was cultured in DMEM/High Glucose (HyClone, SH30243) supplemented with 10% fetal bovine serum (BI, #04–001-1ACS).

### Differential analysis and gene set enrichment analysis

Differential gene expression analysis of MIR4435-2HG and USF1 was conducted based on the Cancer Genome Atlas (TCGA) (https://www.cancer.gov/about-nci/organization/ccg/Research/structural-genomics/tcga) dataset using the R package DESeq2. In our study, Gene Set Enrichment Analysis (GSEA) was performed on the differential expression genes using the R package clusterProfiler (3.8.0) [12]. In GSEA analysis, values of p.adj < 0.05 and false discovery rate (FDR) < 0.25 were considered statistically significant.

### TRRUST database

Transcriptive Regulatory Relationships Unrecovered by Sentence based Text Mining (TRRUST) database (https://www.grnpedia.org/trrust) is a database containing human and mouse transcriptional regulatory networks. The data in TRRUST comes from 112,337 articles retrieved from PubMed, and the database also provides information on the regulatory mechanisms of transcriptional activation or inhibition [13]. In this study, we mainly used the module of Find key regulators for query genes to predict candidate transcription factors.

### RPISeq database

RNA-Protein Interaction Prediction (RPISeq)(http://pridb.gdcb.iastate.edu/RPISeq/index. html) belongs to the machine learning classifier family, which only uses sequence information to predict RNA protein interactions. RPISeq prediction is based on random forest (RF) or Support Vector Machine (SVM) classifier.

### RNA isolation and reverse transcription qPCR (RT-qPCR)

Samples were homogenized in Trizol (Invitrogen; Thermo Fisher Scientific, Inc.) followed by centrifugation at 12,000 rpm for 5 min at 4°C. After extraction of total RNA, reverse transcription was performed with SuperScript III Reverse Transcriptase kit (Thermo Fisher Scientific, Inc.) followed by quantitative qPCR using PowerUp SYBR Green Master Mix (Thermo Fisher). The primer sequences were listed in Supplementary Table S1.

### Western blot

Western blot was conducted as we previously reported [14]. SDS-polyacrylamide gels were made using the PAGE Gel Fast Preparation Kit (Epizyme Biomedical Technology, PG112). All primary antibodies were incubated over-night at 4°C: E-cadherin (Proteintech, 20,874–1-AP), USF1(Proteintech, 22,327–1-AP), CREB1 (Proteintech, 12,208–1-AP), Anti-Succinyllysine (PTM BIO, PTM-419) and AKT (Proteintech, 10,176–2-AP) were used in a 1:1000 dilution, N-cadherin (Proteintech, 22,018–1-AP) was used in a 1:2000 dilution. While Slug (CST, 9585T) and Snail (CST, 3879T) and AKT Ser473 (CST, 4051T) were used in a 1:500 dilution. Monoclonal anti-β-actin (Proteintech, 66,009–1-Ig) was used as endogenous control at a 1:3000 dilution. HRP-conjugated Affinipure Goat Anti-Mouse IgG(H+L) (SA00001-1) and anti-rabbit antibodies HRP-conjugated Affinipure Goat Anti-Rabbit IgG(H+L) (SA00001-2) were purchased from Proteintech and incubated for 1 hour at room temperature. For IP experiments, VeriBlot for IP Detection (Abcam, ab131366) was used at 1:200 for 2 hours at room temperature to enable the trouble-free detection of immunoblotted target protein bands, without interference from denatured IgG.

### Cell counting Kit-8 (CCK8) assay

1.5 × 10^3^ tumor cells were planted in clear bottom 96-well plates and the cell proliferation assays were performed using CCK8 colorimetric assay (Dojindo Molecular Technologies), according to manufacturer’s instructions. The optical density (OD) was measured by a spectrophotometer at 450 nm.

### Plate colony formation assay

3 × 10^3^ tumor cells were planted in clear bottom 6-well plates and incubated for about 10 days until colony formation was observed. Then the culture medium was discarded and the cells were gently washed with PBS. Colonies were fixed in 4% neutral-buffered formaldehyde and stained for 15 min using a crystal violet staining solution (0.25% crystal violet). Each experimental group was set up in three replicates.

### Scratch wound-healing assay

The scratch wound-healing assay was performed to determine the cell migration. HNSCC cells were seeded in 6-well plates one day before the experiment at a density that reached 100% confluency on the following day. Wound healing scratches were imaged immediately after the initial scratch time. Then, the tumor cells were cultured in serum-free culture medium and imaged the scratch again to compare the changes in scratch width between the experimental and control groups.

### Transwell migration and invasion assay

Cell migration and invasion assays were performed with Corning Transwell chamber (8-μm pore size). 4 × 10^4^ tumor cells were planted in the upper chamber with 5% FBS medium and 25% FBS medium was added in the lower chambers. As for the invasion assay, the Transwell chambers were coated with Matrigel Basement Membrane Matrix (BD) and placed in the 37°C cell incubator for 30 min before cell seeding. After 24 hours of culture, the number of tumor cells that migrated through the Transwell membrane was calculated by imaging the lower chamber of the Transwell plate using brightfield microscopy.

### Cell transfection and lentivirus infection

Tumor cells were seeded in six-well plates with 80–90% confluency. All the cell transfections used Lipofectamine 3000 (Invitrogen) in accordance with manufacturer protocols with 500 ng plasmid contained in each test mixture. And the tumor cells were collected 48 hours after the transfection for subsequent experiments. As for lentivirus infection, tumor cells were seeded in six-well plates with 50% confluency. The multiplicity of infection (MOI) was calculated by dividing the number of virus particles by the cell number during infection. Next, cells infected with MIR4435-2HG- overexpressing lentivirus were selected with 2.5 μg/ml Puromycin (Sigma-Aldrich, Cat#: P9620) 48 hours after infection.

### Dual luciferase reporter assay

The MIR4435-2HG promoter plasmid, CREB1 plasmid, and control group plasmid were transfected into tumor cells of the experimental and control groups respectively. MIR4435-2HG promoter and CREB1 plasmid encoded the firefly luciferase gene and the pGL4.13 empty vector was used as an internal control. As the reference control, the pRL-TK plasmid was routinely co-transfected to all samples. Afterward, Firefly and Renilla luciferase activities were measured 36 hours post transfection using the Dual-Luciferase® Reporter Assay System (Promega).

### RNA binding protein immunoprecipitation assay (RIP)

RIP assays were performed using the RNA Immunoprecipitation Kit (Geneseed) following the manufacturer’s instructions. We first extract cell samples according to the protocol. At this point, 10% of the sample was set aside to serve as the input control. After the protein A/G magnetic beads are pretreated, antibodies are added and incubated together for 2 hours. Then the cell samples were added afterwards to incubate together at 4°C overnight on a rotator to capture the antibodies on magnetic beads, and sequential washing was conducted to remove unbound antibodies. Subsequently, RNA was extracted using DR Columns and RC Columns in the kit. The obtained RNA products were analyzed by RT-qPCR, as described previously.

### Immunohistochemistry

HNSCC cells were seeded on coverslips in 24-well plate and cultured overnight. Then the cells were washed with PBS, fixed with 4% formaldehyde solution for 30 minutes at room temperature. Next, the slides were permeabilized with 0.4% Triton X-100 and then blocked with 5% goat serum in BSA–0.4% Triton for 30 minutes. This was followed by incubation with primary antibodies in aforementioned blocking liquid at 4°C overnight, then secondary antibodies Alexa Fluor® 488 (Abcam, ab150077) in blocking liquid for 1 hour at room temperature, and finally after the final wash with PBST, the slides were sealed with anti-fluorescence quenching sealing tablets and observed under a fluorescence microscope to collect images.

### RNA in situ hybridization

RNA in situ hybridization was performed according to the manufacturer’s recommendations (RiboTM Fluorescent In Situ Hybridization Kit, RN: R11060.10). Specifically, HNSCC cells were seeded on coverslips, washed with PBS, fixed with 4% formaldehyde solution, then permeabilized with a solution of 0.5% Triton X-100 in PBS at 4°C for 5 min. The samples were then pre-incubated in hybridization buffer at 37°C for 30 minutes before overnight incubation, at 37°C, in hybridization buffer containing Cy3-labelled MIR4435-2HG RNA probes. After washing, sections were stained with DAPI, imaged on the Zeiss LSM 880 confocal microscope and analyzed using the Zen 2011 software.

### Animal experiments

Mice were housed under specific pathogen-free conditions with a 12 h light/dark cycle,

controlled temperature (22 ± 2°C) and humidity (50–60%), and had ad libitum access to food and water. Mice were purchased at 6 weeks of age from GemPharmatech and allowed to acclimatize to the laboratory environment before experimentation. All experimental procedures were initiated when the animals reached 7 weeks of age. No statistical methods were used to predetermine sample size. 10 male BALB/c Nude mice were randomly divided into 2 groups: control and experimental groups (*n* = 5 in each group), and separately injected with SNU899-Vector and SNU899-MIR4435-2HG overexpressing cells via tail vein injection. The mice were sacrificed at 2 months post injection, and lung tissues were formalin-fixed, paraffin-embedded, sectioned, and stained with haematoxylin and eosin (H&E) for analysis of metastatic burden. Mice were anaesthetised by intraperitoneal injection of 1% sodium pentobarbital. After the animals were confirmed to be in a state of deep anaesthesia, cervical dislocation was performed for euthanasia. Investigators were not blinded to group allocation during data collection and analysis. All procedures were conducted in accordance with the American Veterinary Medical Association (AVMA) Guidelines for the Euthanasia of Animals. All animal experiments were approved by the Institutional Animal Care and Use Committee of Sun Yat-Sen University. The study was reviewed and approved under ethical application number 2,024,002,055.

### Statistical analysis

For TCGA data analysis, statistical tests were performed using the R package stats. In addition, GraphPad Prism 5 was used to conduct statistical analysis. Paired t-test was performed for comparisons between two groups when the data were normally distributed, or Wilcoxon signed-rank tests for those with a non-normal distribution. Values of two-sided *p* ≤ 0.05 were considered statistically significant. All experiments were independently repeated at least three times. For in vitro assays, three independent biological replicates were performed, with each condition analyzed in technical triplicates. Western blot experiments were repeated at least three times with consistent results. *p < 0.05, **p < 0.01, ***p < 0.001.

## Results

### MIR4435-2HG is highly expressed in HNSCC and is associated with poor outcome

**Figure 1.**
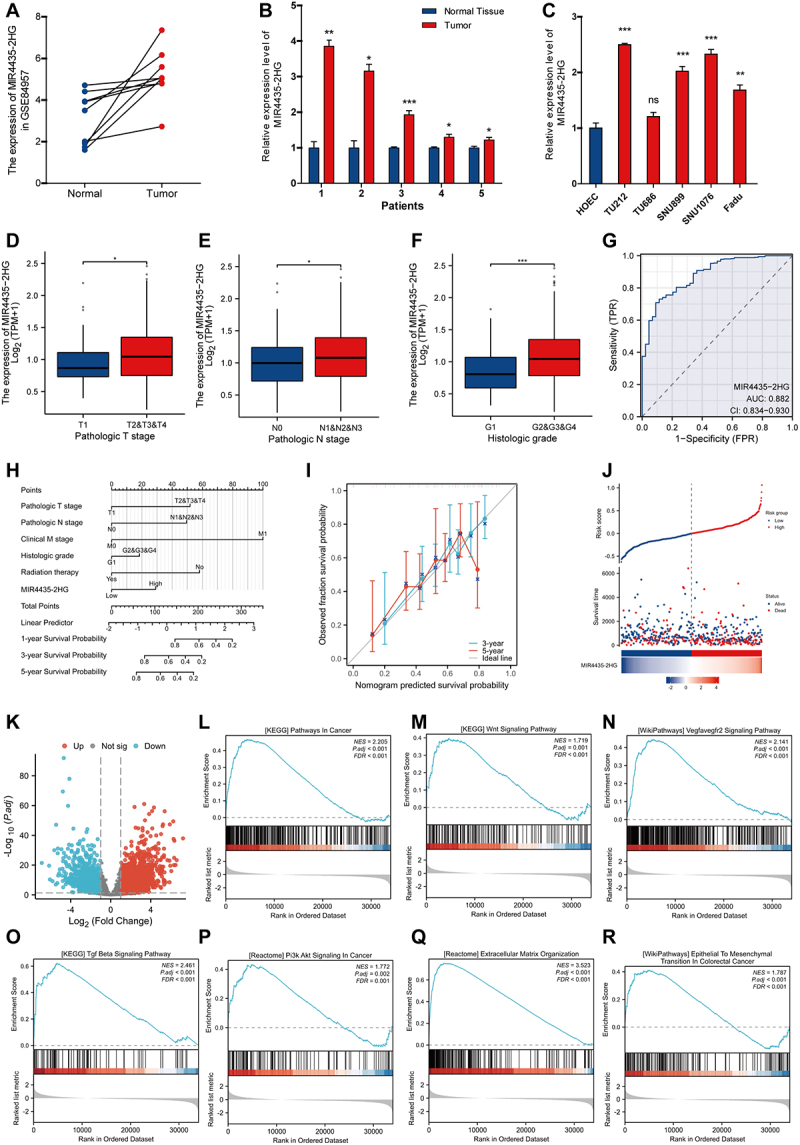
Differential expression analysis of MIR4435-2HG in HNSCC and the analysis of clinicopathological characteristics and the construction of prognostic models. (A) The expression of MIR4435-2HG in laryngeal squamous cell carcinoma tumor tissue was significantly higher than that in adjacent normal control tissue in the GSE84957 dataset. (B) The expression of MIR4435-2HG in fresh samples of 5 cases of laryngeal squamous cell carcinoma was significantly higher in tumor tissues than in adjacent normal control tissues. (C) The expression of MIR4435-2HG in the HNSCC cell line was significantly higher than that in normal endothelial cells. (D) The expression of MIR4435-2HG is associated with pathological T staging. (E) The correlation between the expression of MIR4435-2HG and pathological N staging. (F) The expression of MIR4435-2HG is correlated with pathological grading. (G) The ROC curve analysis shows that the area under the curve is 0.882. (H) Nomogram column chart. (I) Calibration curve with predicted time of 3 and 5 years respectively. (J) Risk factor map. (K) Volcano plot of differentially expressed genes of MIR4435-2HG in TCGA-HNSC. (L-R) GSEA enrichment analysis shows that MIR4435-2HG is involved in regulating pathways in cancer (L), Wnt signaling pathway (M), VEGFR signaling pathway (N), TGF-β signaling pathway (O), PI3K AKT signaling pathway (P), extracellular matrix organization (Q), Ecm receptor interaction (r). * *p* < 0.05, **p < 0.01, ***p < 0.001.

Differential expression analysis of the GSE84957 dataset revealed that MIR4435-2HG expression was significantly elevated in laryngeal squamous cell carcinoma (LSCC) tissues compared with matched adjacent normal tissues (Figure 1(A)). This finding was further validated in an independent cohort of five paired fresh LSCC tumor and adjacent non-tumor tissues, which showed a consistent upregulation of MIR4435-2HG in tumor samples (Figure 1(B)). In line with these observations, analysis of public databases confirmed aberrant overexpression of MIR4435-2HG in HNSCC. Moreover, MIR4435-2HG expression was markedly higher in HNSCC cell lines (TU212, SNU899, SNU1076, and FaDu) than in normal human oral epithelial cells (HOEC) (Figure 1(C)).

To explore the clinical relevance of MIR4435-2HG expression, we next examined its association with clinicopathological parameters in patients with HNSCC. Elevated MIR4435-2HG expression was significantly correlated with advanced pathological T stage, lymph node involvement (N stage), and higher histological grade (Figures 1(D–F)). In contrast, no statistically significant association was observed with distant metastasis (M stage) (Supplementary Figure S1A). Receiver operating characteristic (ROC) curve analysis demonstrated a favourable predictive performance of MIR4435-2HG expression for HNSCC outcomes, with an area under the curve (AUC) of 0.882 (Figure 1(G)). Survival analyses further indicated that patients with high MIR4435-2HG expression had significantly poorer overall survival compared with those in the low-expression group (*p* = 0.002, log-rank test; Supplementary Figure S1B). Multivariate Cox regression analysis identified MIR4435-2HG expression as an independent prognostic factor for HNSCC (Supplementary Figure S2). Based on these variables, a prognostic nomogram was constructed to estimate individual patient outcome probabilities (Figure 1(H)). Calibration curve analysis demonstrated good agreement between predicted and observed outcomes, indicating satisfactory predictive accuracy of the model (Figure 1(I)). Consistently, the risk factor plot showed a progressive increase in predicted risk and mortality with higher MIR4435-2HG expression levels (Figure 1(J)).

Collectively, these results demonstrate that MIR4435-2HG is markedly upregulated in HNSCC and is closely associated with aggressive clinicopathological features and unfavourable patient prognosis.

### Enrichment analysis identifies MIR4435-2HG – associated signaling pathways and invasive programs in HNSCC

We divided the patients into high and low expression groups based on MIR4435-2HG expression in the TCGA-HNSC database to further clarify the molecular mechanism of MIR4435-2HG in the progression of HNSCC. The results were shown in the volcano map (Figure 1(K)), with 680 up-regulated and 1051 down-regulated expression groups showing statistical differences. Gene set enrichment analysis (GSEA) revealed that MIR4435-2HG – associated gene signatures were significantly enriched in multiple cancer-related signalling pathways (Figure 1(L)). Notably, pathways implicated in tumor growth, angiogenesis, and invasion were prominently represented, including the Wnt signalling pathway (Figure 1(M)), VEGFR signalling pathway (Figure 1(N)), TGF-β signalling pathway (Figure 1(O)), and PI3K – AKT signalling pathway (Figure 1(P)). In addition to canonical oncogenic signalling, enrichment analyses also highlighted biological processes related to tumor cell motility, invasion, metastasis, and EMT (Figures 1(Q,R)). Collectively, these enrichment profiles suggest that elevated MIR4435-2HG expression is closely linked to activation of multiple pro-tumorigenic pathways and invasive programmes in HNSCC, providing a mechanistic basis for its association with aggressive tumor behaviour observed in clinical analyses.

### MIR4435-2HG promotes invasion and metastasis of HNSCC cells by activating the AKT signaling pathway

**Figure 2.**
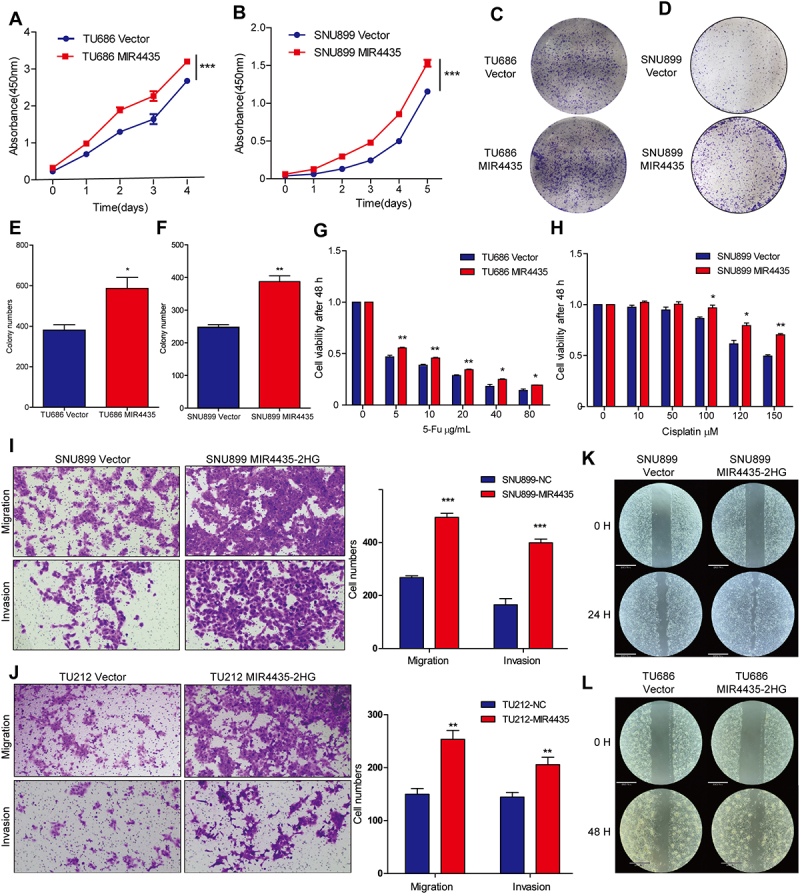
MIR4435-2HG promotes malignant behaviors of HNSCC cells. (A-B) CCK8 experiment for proliferation. (C-F) The plate colony formation assay and the statistical analysis of the number of plate clones formed. (G-H) Drug resistance experiments of head and neck squamous cell carcinoma cells to 5-fu and cisplatin. (I-J) Transwell assay analyses of the migration and invasion abilities of the SNU899 and TU212 cells. (K-L) The scratch wound healing assay.

**Figure 3.**
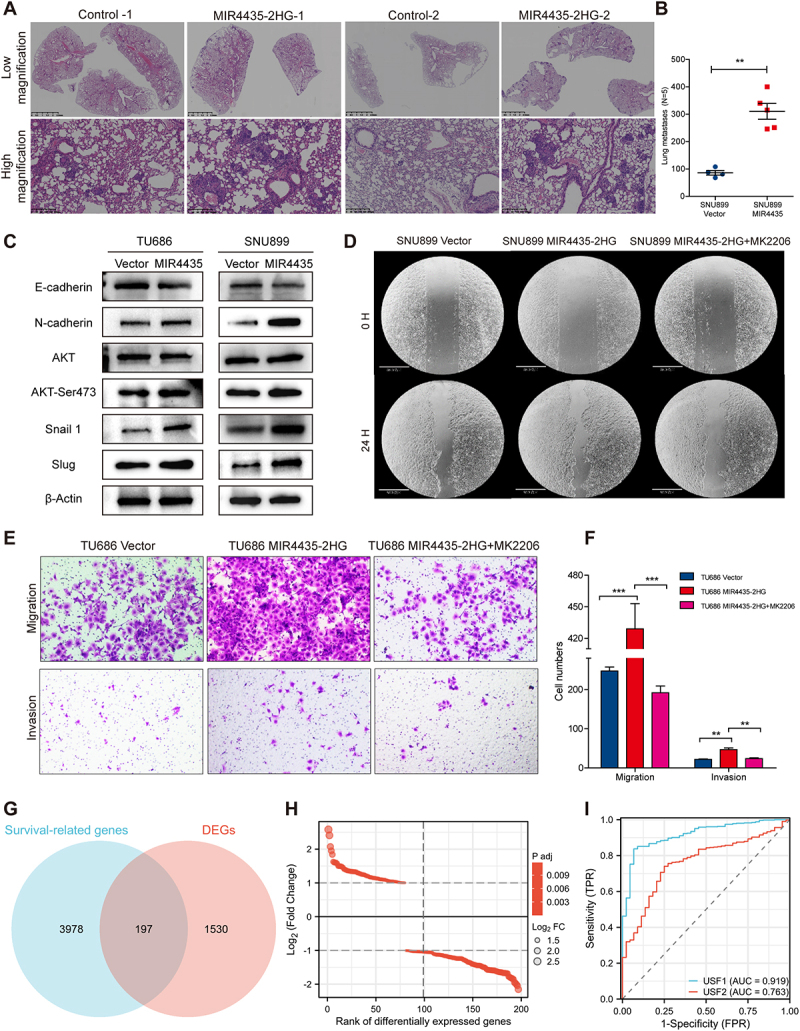
MIR4435-2HG promotes invasion and metastasis of HNSCC cells by activating the AKT signaling pathway. (A) H&E staining of lung sections from nude mice that were tail-vein injected with HNSCC cells. (B) Number of lung metastases was counted under ithe microscope. (C) Western-Blot analysis of EMT-related biomarkers after overexpression of MIR4435-2HG. (D) The mobility of cells was measured by testing the rate of wound closure at 0 and 24 hours with overexpression of MIR4435-2HG and treatment of MK2206. (E) The migratory and invasion capacity of HNSCC were tested in a transwell migration assay. (F) The graph showed the results of migration and invasion assay presented as means ± SD. (G) by using the Wayne plot, 197 intersecting genes were obtained from the survival related genes dataset (blue) and MIR4435-2HG differentially expressed genes dataset (DEGs) (red) in the TCGA-HNSC database. (H) Volcano map of 197 intersecting gene differences in the Wayne diagram. (I) The ROC analysis of USF1 and USF2 in TCGA-HNSC. **p < 0.05, ***p < 0.01, *p < 0.001.

As indicated by previous GSEA enrichment analysis, MIR4435-2HG is implicated in regulating tumor invasion, metastasis, and the AKT signaling pathway in HNSCC. To further investigate its functional role, we assessed cell proliferation using the CCK8 assay over a 5-day period. HNSCC cells overexpressing MIR4435-2HG exhibited a significantly enhanced proliferation rate compared with the control group (Figure 2(A,B)). Additionally, plate colony formation assays showed a marked increase in the number of colonies in MIR4435-2HG-overexpressing cells (Figure 2(C–F)). Notably, overexpression of MIR4435-2HG also conferred resistance to chemotherapy agents, including 5-fluorouracil and cisplatin (Figure 2(G,H)). The migratory and invasive capacities of the MIR4435-2HG overexpressing cells were further assessed using transwell assays, which revealed a significant increase in the number of tumor cells migrating and invading through the chamber (Figure 2(I,J)). In the scratch wound healing assay, MIR4435-2HG overexpression markedly enhanced cell migration (Figure 2(K,L)). To validate these findings in vivo, a lung metastasis model was established by tail vein injection of the aforementioned HNSCC cells into nude mice. Haematoxylin and eosin (H&E) staining of the lung tissues revealed significantly increased metastatic foci in the experimental group compared to the scramble control (Figure 3A,B).

We next examined the expression of EMT markers following MIR4435-2HG overexpression. Notably, we observed a downregulation of the epithelial marker E-cadherin and upregulation of the mesenchymal marker N-cadherin (Figure 3(C)). Moreover, the EMT-related transcription factors Slug and Snail were significantly upregulated in MIR4435-2HG-overexpressing cells. Along with these changes, we found that phosphorylation of AKT (Ser473) was markedly elevated upon MIR4435-2HG overexpression (Figure 3(C)), suggesting activation of the AKT signalling pathway. To investigate the dependency on AKT signalling, MIR4435-2HG-overexpressing HNSCC cells were treated with the AKT inhibitor MK2206 (10 μM). As anticipated, MK2206 treatment partially reversed the enhanced migration observed in the scratch wound healing assay (Figure 3(D)). Furthermore, significant reductions in cell migration and invasion were observed following AKT inhibition (Figure 3(E,F)).

In summary, these findings demonstrate that MIR4435-2HG promotes invasion and metastasis of HNSCC cells by activating the AKT signalling pathway, suggesting that targeting MIR4435-2HG and its downstream signalling axis could be a promising therapeutic strategy for HNSCC.

### Screening of the downstream core transcription factor USF1

**Figure 4.**
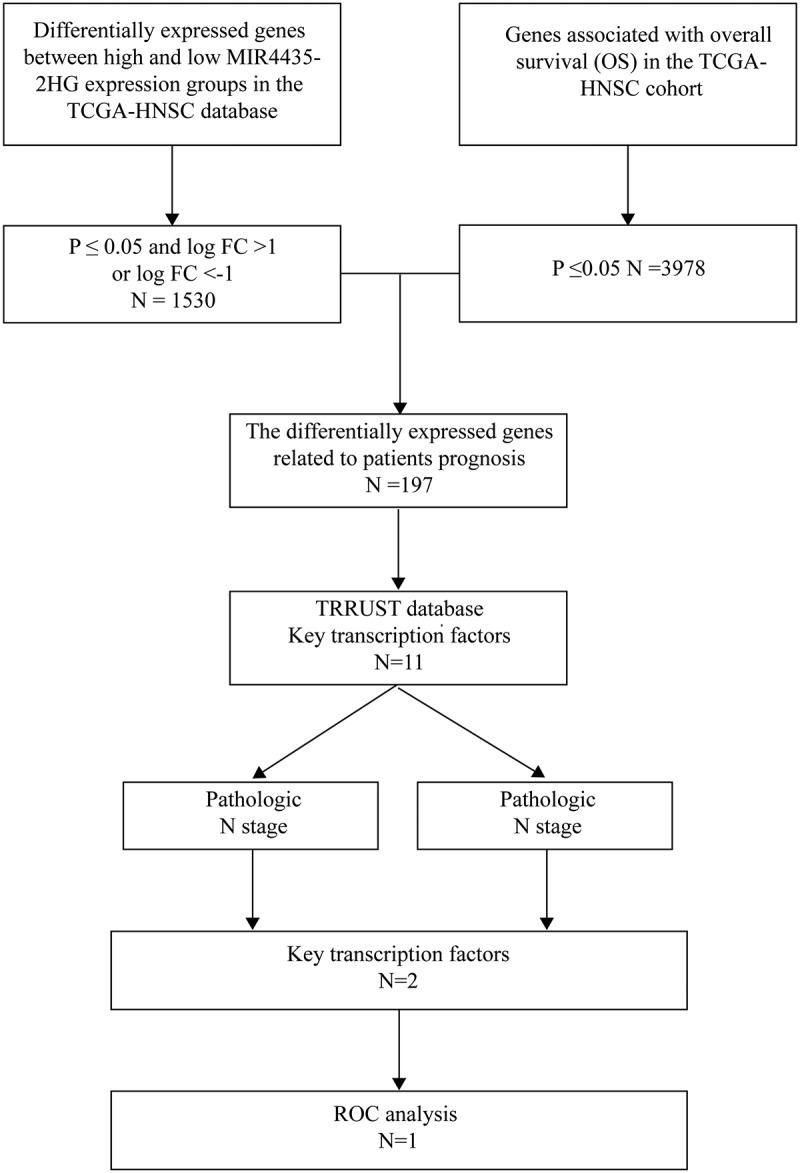
The screening process was summarized in the study flow diagram.

To identify the specific downstream regulatory molecules of MIR4435-2HG, we conducted a systematic screening, as illustrated in the study flow diagram (Figure 4). Initially, survival-associated genes related to overall survival (OS) in HNSCC patients were retrieved from the TCGA database, yielding a total of 3,978 genes. Subsequently, differentially expressed genes (DEGs) associated with MIR4435-2HG were selected based on criteria of *p* ≤ 0.05 and |logFC| >1, resulting in 1,530 DEGs in the TCGA-HNSC dataset. The intersection of these DEGs with the survival-related genes was then visualized using a Venn diagram, revealing 197 genes that were both differentially expressed and correlated with patient prognosis (Figure 3(G)), of which 117 were downregulated and 80 were upregulated. The volcano plot of these differential genes is shown in Figure 3(H). Further investigation was performed to identify the core transcription factors regulating these 197 genes. Using the TRRUST online database, we identified 11 potential transcription factors, including PAX2, Snail1, Twist1, Twist2, USF1, USF2, TP53, EGR1, RELA, NFKB1, and SP1 (Table 1). To evaluate the binding probability of these transcription factors to MIR4435-2HG, we used the RPISeq online tool, which predicted high binding affinity (with RF and SVM values both greater than 0.5) for several transcription factors, as shown in Table 1.

**Table 1.** TRRUST database analysis of key transcription factors.

Key TF	*p* value	FDR	Prediction using RF classifier	Prediction using SVM classifier	*p* Value of pathologic N stage	*p* Value of Lymphovascular invasion
PAX2	0.000602	0.00662	0.85	0.95	NA	**
Snail1	0.00177	0.00976	0.90	0.92	*	NA
Twist1	0.0111	0.0407	0.90	0.88	**	NA
Twist2	0.0211	0.0581	0.90	0.93	NA	NA
USF2	0.0365	0.0804	0.80	0.89	**	**
USF1	0.0653	0.12	0.80	0.85	***	***
TP53	0.0886	0.139	0.90	0.96	NA	NA
EGR1	0.11	0.151	0.90	0.91	NA	NA
RELA	0.304	0.338	0.90	0.93	NA	NA
NFKB1	0.308	0.338	0.80	0.93	NA	NA
SP1	0.586	0.586	0.90	0.90	NA	NA

Key TF: Key transcription factor; NA: No statistical difference *p < 0.05, **p < 0.01, ***p < 0.001.

Given the known association of MIR4435-2HG with HNSCC invasion and metastasis, we further analysed the correlation between these 11 transcription factors and clinical parameters, such as pathological N stage and lymphatic invasion, in the TCGA-HNSC cohort. The results revealed that only USF1 and USF2 exhibited statistically significant associations with both pathological N stage and lymphatic invasion (Table 1, Supplementary Figures S1C – S1M, S2A – S2K). Among these, USF1 showed superior diagnostic performance for predicting patient outcomes, with an area under the curve (AUC) of 0.919, compared to an AUC of 0.763 for USF2 (Figure 3(I)).

### MIR4435-2HG promotes tumor invasion and metastasis by regulating the protein expression levels of USF1 in HNSCC

**Figure 5.**
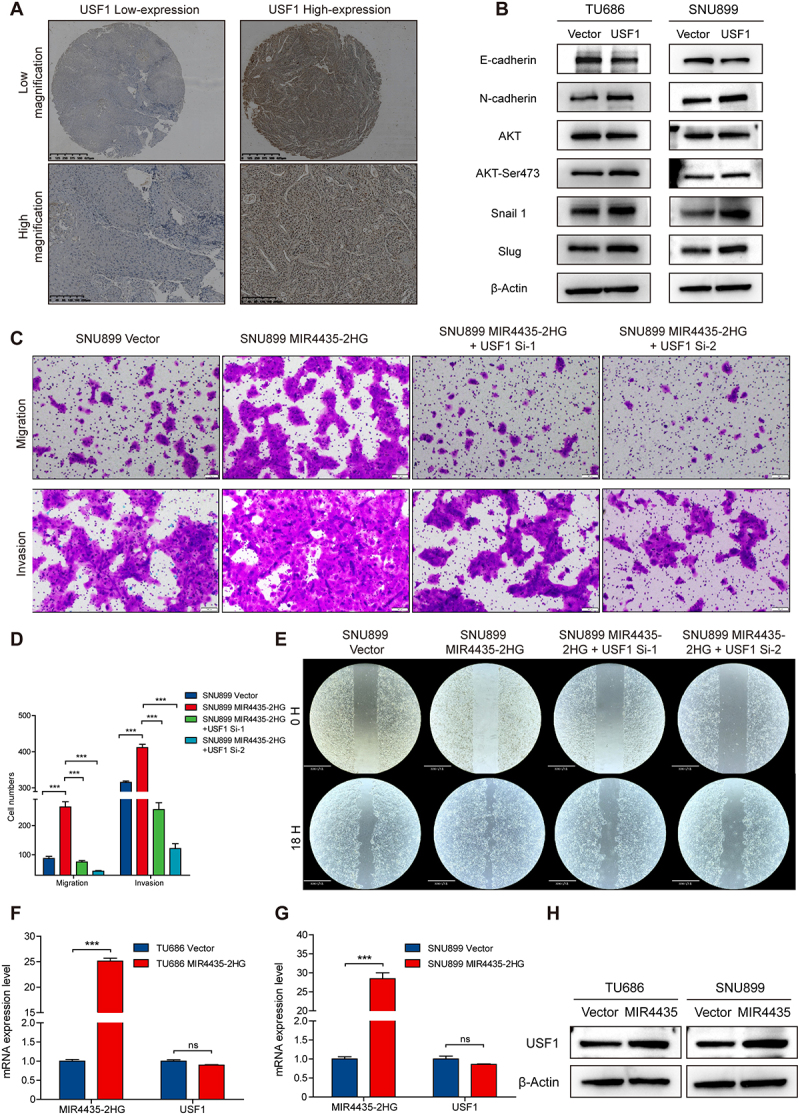
LncRNA MIR4435-2HG promotes HNSCC cell invasion and metastasis by regulating the expression of USF1. (A) Immunohistochemical staining revealed variable expression levels of USF1 protein in patients with head and neck squamous cell carcinoma. (B) The Western Blot experiment results showed that USF1 could activate the AKT signalling pathway and promote the EMT of HNSCC cells. (C) The migratory and invasion capacity of HNSCC were tested in a transwell migration assay. (D) The graph showed the results of migration and invasion assay presented as means ± sd. (E) The mobility of cells was measured by testing the rate of wound closure at 0 and 18 hours with overexpression of MIR4435-2HG and knock down of USF1. (F-G) The results of RT-qPCR analysis showed that there was no significant change in the expression level of USF1 mRNA in TU686 and SNU899 cells overexpressing MIR4435-2HG. (H) the Western Blot experiment results showed that overexpression of MIR4435-2HG significantly upregulated the expression level of USF1 protein in TU686 and SNU 899. **p < 0.05, ***p < 0.01, *p < 0.001.

A tissue microarray consisting of 75 formalin-fixed paraffin-embedded (FFPE) HNSCC samples was constructed, and immunohistochemical (IHC) staining was performed as previously described. The correlation between USF1 protein expression and clinicopathological features was analysed, revealing that elevated USF1 expression was significantly associated with histological differentiation and lymph node metastasis (Table 2, Figure 5(A)). Consistent with these findings, USF1 overexpression promoted epithelial – mesenchymal transition (EMT) through activation of the AKT signalling pathway (Figure 5(B)).

**Table 2.** The correlation of USF1 expression with clinical characteristics of HNSCC patients, 75 cases.

Clinical information (%)		USF1-Low *N* = 24	USF1-High *N* = 51	*p* value
Gender	Male	24 (32%)	49 (65.3%)	0.830
	Female	0 (0%)	2 (2.7%)	
Age(years)	<62	8 (10.7%)	29 (38.7%)	0.057
	≥62	16 (21.3%)	22 (29.3%)	
Differentiation	Medium-High	13 (17.3%)	12 (16%)	0.017
	Medium	6 (8%)	13 (17.3%)	
	Medium-Low	5 (6.7%)	26 (34.7%)	
Lymph node metastasis (N)	No	19 (25.3%)	27 (36%)	0.030
	Yes	5 (6.7%)	24 (32%)	
Diameter (cm)	<2.5	13 (17.3%)	24 (32%)	0.566
	≥2.5	11 (14.7%)	27 (36%)	

To investigate whether the effects of MIR4435-2HG on tumor invasion and metastasis are dependent on USF1 expression, we generated HNSCC cell lines with USF1 knockdown using siRNA and performed rescue experiments. As anticipated, MIR4435-2HG overexpression significantly increased the number of migrating and invading cells in the transwell assay. However, simultaneous knockdown of USF1 led to a significant reduction in the number of tumor cells that passed through the transwell membrane (Figure 5(C)), and these differences were statistically significant (Figure 5(D)). Similarly, MIR4435-2HG overexpression enhanced the wound healing ability in the scratch assay, a phenotype that was partially rescued by USF1 knockdown (Figure 5(E)). Taken together, these results suggest that the invasive and metastatic potential of HNSCC cells promoted by MIR4435-2HG is dependent on USF1 expression.

### MIR4435-2HG enhances protein stability by promoting succinylation via its interaction with USF1

**Figure 6.**
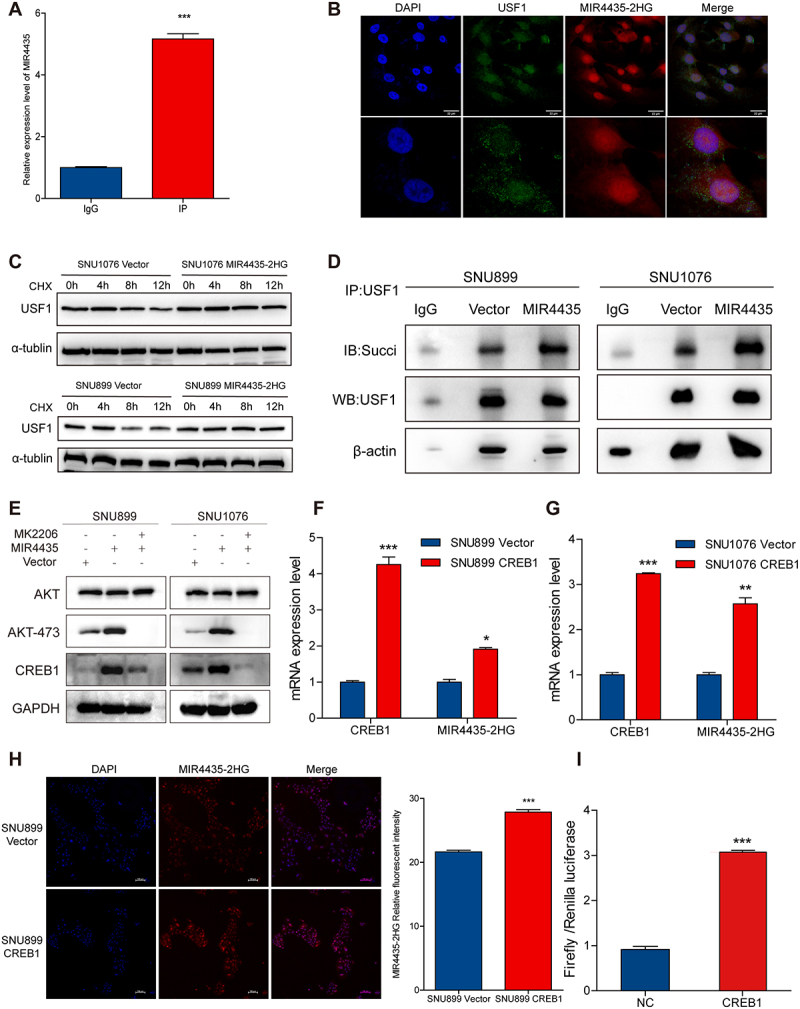
MIR4435-2HG promotes USF1 succinylation and forms a feedback loop with CREB1. (A) The RIP experiment results showed that the expression level of MIR4435-2HG was significantly upregulated in the USF1-IP group compared to the IgG group. (B) The results of laser confocal scanning showed that USF1 (Green) and LncRNA MIR4435-2HG (Red) were co-localized. (C) Western blot to detect USF1 protein levels after cycloheximide (CHX) treatment. (D) IP, Western blot assay to detect USF1 succinylation levels following MIR4435-2HG overexpression. (E) Western blot assay to verify that MIR4434-2HG promotes the expression of CREB1 protein by activating the AKT signaling pathway. (F-G) RT-qPCR detection showed that overexpression of USF1 upregulated the expression level of downstream target gene MIR4435-2HG. (H) The expression of MIR4435-2HG was detected and quantified by immunofluorescence in the head and neck squamous cell carcinoma cell line overexpressing CREB1. (I) In the double Luciferase report experiment, the ratio of Firefly/Renilla luciferase in the CREB1 overexpression experiment group was significantly increased. **p < 0.05, ***p < 0.01, *p < .001, ns means no statistically significant difference. **p < 0.05, ***p < 0.01, *p < 0.001.

To further elucidate the regulatory mechanism by which MIR4435-2HG influences USF1 expression, we examined the protein and mRNA levels of USF1 following MIR4435-2HG overexpression. Notably, MIR4435-2HG upregulated USF1 at the protein level but did not affect its mRNA expression (Figures 5(F,G)), suggesting that MIR4435-2HG may regulate USF1 through post-translational modifications. The predicted high binding affinity between MIR4435-2HG and USF1 (Table 1) led us to hypothesize that MIR4435-2HG stabilizes USF1 by binding to it. RNA immunoprecipitation (RIP) experiments demonstrated that MIR4435-2HG was significantly enriched in the immunoprecipitated samples compared to the control IgG group (Figure 6(A)). Confocal immunofluorescence analysis revealed a strong nuclear co-localization of MIR4435-2HG and USF1, supporting their physical interaction (Figure 6(B)).

To confirm that MIR4435-2HG regulates USF1 stability, we treated transfected HNSCC cells with cycloheximide (CHX) to inhibit protein synthesis. The results showed that MIR4435-2HG overexpression significantly enhanced USF1 protein stability (Figure 6(C)). Furthermore, we observed increased succinylation of USF1 in cells overexpressing MIR4435-2HG, as indicated by immunoprecipitation with magnetic beads and subsequent analysis of succinylation levels (Figure 6(D)). These findings suggest that MIR4435-2HG enhances USF1 stability by promoting its succinylation, thereby facilitating the invasion and metastasis of HNSCC cells.

### CREB1 promotes the expression of target gene MIR4435-2HG

Studies have confirmed that AKT can promote the phosphorylation of CREB1 and activate its transcriptional activity [15,16]. Given the involvement of CREB1 in the proposed regulatory axis, we further assessed its clinical relevance in the TCGA-HNSC cohort. CREB1 expression was higher in tumor tissues than in normal tissues (Supplementary Figure S3A-S3B) and showed an association with histologic grade (Supplementary Figure S3C-S3F). Kaplan – Meier analysis did not demonstrate a significant relationship between CREB1 expression and overall or disease-specific survival (Supplementary Figure S3G-S3I). In Cox regression analysis for progression-free interval (PFI), CREB1 was not significant in univariate analysis (*p* = 0.102), whereas it reached significance in the multivariate model (*p* = 0.035) (Supplementary Figure S3). ROC analysis yielded an AUC of 0.757, indicating moderate discriminative ability (Supplementary Figure S3J). In our experiments, increased AKT phosphorylation was associated with elevated CREB1 expression levels, whereas treatment with the PI3K/AKT pathway inhibitor MK2206 resulted in a significant decrease in CREB1 expression (Figure 6(E)). To investigate the impact of CREB1 on MIR4435-2HG expression, we performed RT-qPCR and fluorescent in situ hybridization (FISH) assays. These analyses revealed that overexpression of CREB1 led to upregulation of MIR4435-2HG expression (Figures 6(F–H)). Furthermore, we employed a dual luciferase reporter assay in HEK 293T cells to assess the effect of CREB1 on transcriptional activation. The results demonstrated a significant increase in the firefly-to-Renilla luciferase activity ratio 36 hours after CREB1 overexpression, indicating enhanced transcriptional activity (Figure 6(I)). Together, these findings suggest that CREB1 acts as a transcriptional activator of MIR4435-2HG, thereby forming a positive feedback loop that amplifies the cancer-promoting effects of MIR4435-2HG.

## Discussion

LncRNA MIR4435-2HG, also known by various names including MIR4434-1HG, LINC00978, AK001796, lncRNA-AWPPH, AGD2, or MORRBID, is primarily located at band 3 of region 2 on the long arm of chromosome 2. Initially identified by Yiguo Jiang in 2015, MIR4435-2HG was found to be highly expressed in lung cancer tissues and cell lines, where it was shown to be involved in the anticancer effects of resveratrol [8]. Subsequently, the lncRNA has been reported to be overexpressed in various tumor types, including leukaemia, bladder cancer, nasopharyngeal cancer, lung cancer, liver cancer, melanoma, glioma, colorectal cancer, oral squamous cell carcinoma, and oesophageal cancer, among others [17]. In these cancers, MIR4435-2HG generally exerts pro-tumorigenic roles, influencing key signalling pathways such as Wnt/β-catenin, PI3K/AKT, TNFα, Ras/Raf/MEK/ERK, TGF-β, and MDM2/p53 [3,5,18–20].

The molecular mechanisms underlying the effects of MIR4435-2HG have been explored in several studies, with three primary regulatory roles identified: first, as a competing endogenous RNA (ceRNA), MIR4435-2HG sequesters downstream miRNAs, thus functioning as a molecular sponge; second, MIR4435-2HG participates in epigenetic regulation, acting as a scaffold for histone-modifying complexes at target loci; and third, it directly interacts with downstream proteins, influencing their stability and, consequently, their biological functions.

In summary, MIR4435-2HG plays a crucial role in various cancers and holds promise as a biomarker for early diagnosis, monitoring distant metastasis, and assessing patient prognosis. However, research on its role in head and neck squamous cell carcinoma (HNSCC) remains limited. Previous studies have suggested that Fusobacterium nucleatum can induce EMT in both normal and cancerous oral epithelial cells by regulating the MIR4435-2HG/miR-296-5p/Akt2/SNAI1 pathway [21]. Moreover, Luyue Zhang et al. demonstrated that MIR4435-2HG exerts a cancer-promoting effect via TGF-β1 signalling in oral squamous cell carcinoma [22].

In our study, we analysed DEGs in high and low MIR4435-2HG expression groups using the TCGA-HNSC database, followed by gene set enrichment analysis (GSEA) to explore their biological functions in HNSCC. The results indicated that MIR4435-2HG regulates multiple pathways and phenotypes associated with invasion and metastasis, including EMT, activation of matrix metalloproteinases, and extracellular matrix remodelling. These findings led us to hypothesize that MIR4435-2HG promotes EMT in HNSCC, thus enhancing tumor cell invasion and metastasis. To validate this hypothesis, we conducted transwell migration and invasion assays, scratch wound healing assays, and Western blot analyses, all of which confirmed the involvement of MIR4435-2HG in these processes. We further investigated its specific regulatory mechanism by intersecting DEGs with survival-related genes, identifying functional differentially expressed genes, and using the TRUST database, clinicopathological index correlation analysis, and ROC analysis to screen for the downstream core transcription factor USF1. The screening process and findings are summarized in the study flow diagram (Figure 4). In the cohort, MIR4435-2HG expression correlated with lymph node metastasis but not with distant metastasis. This pattern may reflect differences in the biological requirements of these processes. Lymphatic spread is often closely linked to local invasion and epithelial – mesenchymal transition (EMT), both of which were influenced by MIR4435-2HG in our functional assays. By contrast, successful distant metastasis requires additional steps, including survival in circulation and colonization of secondary sites, which may depend on broader or distinct regulatory programmes. The lack of association with distant metastasis may therefore indicate a more prominent role of MIR4435-2HG in early metastatic behaviour. It should also be noted that the number of patients with documented distant metastasis was limited, which may reduce the statistical power to detect such an association.

Tumor metastasis remains the leading cause of cancer-related death, and the regulatory mechanisms driving this process require further elucidation. A growing body of research has emphasized the role of epigenetic pathways in metastasis, with a particular focus on post-translational modifications (PTMs), which play pivotal roles in malignancy. Beyond classic acetylation, recent studies have identified several novel acylation forms, including propionylation, butyrylation, malonic acylation, and succinylation, all of which are crucial for cellular energy regulation and signal transduction [23]. Succinylation, in particular, is an important PTM that induces significant conformational changes in proteins by adding succinic acid groups to lysine residues [24]. Recent research has highlighted the impact of succinylation on protein half-life by regulating structural stability, which can either prolong or shorten protein stability depending on its effect on degradation pathways. For example, in lung adenocarcinoma cells, succinylation of SUCLG2 enhances protein stability, promoting cell proliferation and tumor formation [25]. In contrast, succinylation may also lead to the rapid degradation of certain proteins, reducing their biological activity and potentially inhibiting tumor progression. The succinylation of transcription factors plays a significant role in tumor growth by modulating their stability and transcriptional activity. For example, the succinylation of p53, a critical tumor suppressor, profoundly influences its stability and DNA-binding capacity, thereby modulating its transcriptional efficacy [24].

Our findings show that MIR4435-2HG enhances the protein levels of USF1 without affecting its mRNA expression. Bioinformatics analyses and protein – RNA interaction experiments confirmed direct binding between MIR4435-2HG and USF1. This observation suggests that MIR4435-2HG may regulate USF1 at the post-translational level. Consistent with this, we observed that MIR4435-2HG overexpression led to a marked increase in USF1 succinylation. These results indicate that MIR4435-2HG enhances the succinylation of USF1, thereby stabilizing the protein and promoting tumor invasion and metastasis in HNSCC.

The aberrant expression of CREB1 has been documented in various cancers, including haematopoietic malignancies, non-small cell lung cancer, glioblastoma, breast cancer, and melanoma [26,27]. CREB1 regulates proto-oncogenes such as Cyclin A2, EGR-1, MMP2/9, GSK3A, and non-coding RNAs, influencing cell proliferation, differentiation, apoptosis, angiogenesis, inflammatory responses, and tumorigenesis through pathways such as ERK1/2, PKA, PKC, and CaMKII [28,29]. Zha et al. demonstrated that the loss of PTEN upregulates the AKT1-CREB-PDGFRα signalling cascade, promoting tumorigenesis [30]. Based on these findings, CREB1 has emerged as a potential therapeutic target in cancer treatment [19,31]. In the present study, we show that CREB1 enhances the transcriptional activity of MIR4435-2HG in HNSCC, as confirmed through RT-qPCR and dual luciferase reporter assays. Taken together, our results propose that MIR4435-2HG stabilizes USF1 by promoting its succinylation and induces EMT, thereby facilitating tumor invasion and metastasis. Furthermore, activation of the AKT signalling pathway enhances CREB1 transcriptional activity, which in turn upregulates MIR4435-2HG, amplifying its pro-tumorigenic effects.

From a translational perspective, the identification of MIR4435-2HG as a central regulator of the USF1–AKT – CREB1 axis provides a potential entry point for RNA-targeted therapeutic intervention. Recent advances in small RNA – based strategies have demonstrated the feasibility of selectively silencing oncogenic noncoding RNAs or disrupting their functional interactions in cancer [9–11]. In particular, approaches such as small interfering RNAs (siRNAs), antisense oligonucleotides, and microRNA-based therapeutics have shown encouraging efficacy in preclinical and early clinical settings, especially in malignancies driven by dysregulated RNA networks [9,10]. Given that MIR4435-2HG exerts its oncogenic effects through direct protein interaction and post-translational modulation, targeting this lncRNA may not only suppress its expression but also destabilize downstream signalling cascades, including AKT-dependent pathways. Moreover, emerging evidence suggests that RNA-directed therapies can be effectively combined with conventional treatments to overcome resistance and improve therapeutic specificity in head and neck cancers [11]. Therefore, our findings provide a mechanistic rationale for considering MIR4435-2HG as a candidate target for small RNA – based therapeutic strategies in HNSCC.

## Conclusion

In conclusion, MIR4435-2HG is aberrantly upregulated in HNSCC and correlates with poor prognosis. Mechanistically, MIR4435-2HG promotes invasion and metastasis by interacting with the transcription factor USF1 and enhancing its protein stability through succinylation, highlighting a previously unrecognized lncRNA-mediated epigenetic regulatory mechanism. Stabilized USF1 sustains AKT pathway activation, while AKT-driven CREB1 activation transcriptionally upregulates MIR4435-2HG, forming a positive feedback loop that amplifies oncogenic signalling. Collectively, our findings delineate a MIR4435-2HG – USF1–AKT – CREB1 regulatory axis and underscore the role of lncRNA-controlled post-translational modification in HNSCC progression, providing potential targets for therapeutic intervention.

## Supplementary Material

Supplementary Figure 3.tif

Supplementary figure 1.tif

Supplementary Table 3.docx

Supplementary table 1.docx

Supplementary figure 2.tif

Supplementary table 2.docx

## Data Availability

All data related to this study are presented in the supplementary material.

## References

[1] Sung H, Ferlay J, Siegel RL, et al. Global cancer statistics 2020: GLOBOCAN estimates of incidence and mortality worldwide for 36 cancers in 185 countries. CA Cancer J Clin. 2021;71(3):209–19. doi: 10.3322/caac.21660 33538338

[2] Sharma A, Kansara S, Mahajan M, et al. Long non-coding RNAs orchestrate various molecular and cellular processes by modulating epithelial-mesenchymal transition in head and neck squamous cell carcinoma. Biochim Biophys Acta Mol Basis Dis. 2021;1867(11):166240. doi: 10.1016/j.bbadis.2021.166240 34363933

[3] Akbari Dilmaghani N, Khoshsirat S, Shanaki-Bavarsad M, et al. The contributory role of long non-coding RNAs (lncRNAs) in head and neck cancers: possible biomarkers and therapeutic targets? Eur J Pharmacol. 2021;900:174053. doi: 10.1016/j.ejphar.2021.174053 33766619

[4] Kunieda F, Kiyota N, Tahara M, et al. Randomized phase II/III trial of post-operative chemoradiotherapy comparing 3-weekly cisplatin with weekly cisplatin in high-risk patients with squamous cell carcinoma of head and neck: Japan clinical oncology group study (JCOG1008). Jpn J Clin Oncol. 2014;44(8):770–774. doi: 10.1093/jjco/hyu067 24842866

[5] Noronha V, Joshi A, Patil VM, et al. Once-a-week versus once-every-3-weeks cisplatin chemoradiation for locally advanced head and neck cancer: a phase III randomized noninferiority trial. J Clin Oncol. 2018;36(11):1064–1072. doi: 10.1200/JCO.2017.74.9457 29220295

[6] Pan X, Zheng G, Gao C. LncRNA PVT1: a novel therapeutic target for cancers. Clin Lab. 2018;64(5):655–662. 29739059 10.7754/Clin.Lab.2018.171216

[7] Loganathan T, Doss CG. Non-coding RNAs in human health and disease: potential function as biomarkers and therapeutic targets. Funct Integr Genomics. 2023;23(1):33. doi: 10.1007/s10142-022-00947-4 36625940 PMC9838419

[8] Yang Q, Xu E, Dai J, et al. A novel long noncoding RNA AK001796 acts as an oncogene and is involved in cell growth inhibition by resveratrol in lung cancer. Toxicol Appl Pharmacol. 2015;285(2):79–88. doi: 10.1016/j.taap.2015.04.003 25888808

[9] Thomas P, Selvakumar SC, Preethi KA, et al. Expression profiling of signal transducer and activator of transcription3 in oral squamous cell carcinoma in South Indian population. Minerva Dent Oral Sci. 2024;73(1):37–44. doi: 10.23736/S2724-6329.23.04840-4 37878241

[10] Varshan MS, Selvakumar SC, Preethi KA, et al. MicroRNA-34a-3p and its target tumor necrosis factor-alpha in the regulation of South Indian oral squamous cell carcinoma population. Minerva Dent Oral Sci. 2024;73(5):256–263. doi: 10.23736/S2724-6329.23.04835-0 37878240

[11] Pa K, Usman PPA, Sekar D. OIP5-AS1 expression profiles in different stages of oral squamous cell carcinoma. Arch Oral Biol. 2025;180:106403. 41014896 10.1016/j.archoralbio.2025.106403

[12] Subramanian A, Tamayo P, Mootha VK, et al. Gene set enrichment analysis: a knowledge-based approach for interpreting genome-wide expression profiles. Proc Natl Acad Sci U S A. 2005;102(43):15545–15550. doi: 10.1073/pnas.0506580102 16199517 PMC1239896

[13] Han H, Cho JW, Lee S, et al. TRRUST v2: an expanded reference database of human and mouse transcriptional regulatory interactions. Nucleic Acids Res. 2018;46(D1):D380–D386. doi: 10.1093/nar/gkx1013 29087512 PMC5753191

[14] Wang W, He S, Zhang R, et al. ALDH1A1 maintains the cancer stem-like cells properties of esophageal squamous cell carcinoma by activating the AKT signal pathway and interacting with beta-catenin. Biomed Pharmacother. 2020;125:109940. 32044720 10.1016/j.biopha.2020.109940

[15] Hu M, Liu Z, Lv P, et al. Autophagy and Akt/CREB signalling play an important role in the neuroprotective effect of nimodipine in a rat model of vascular dementia. Behav Brain Res. 2017;325(Pt A):79–86. doi: 10.1016/j.bbr.2016.11.053 27923588

[16] Zuo D, Lin L, Liu Y, et al. Baicalin attenuates ketamine-induced neurotoxicity in the developing rats: involvement of PI3K/Akt and CREB/BDNF/Bcl-2 pathways. Neurotox Res. 2016;30(2):159–172. doi: 10.1007/s12640-016-9611-y 26932180

[17] Guo D, Liu F, Zhang L, et al. Long non-coding RNA AWPPH enhances malignant phenotypes in nasopharyngeal carcinoma via silencing PTEN through interacting with LSD1 and EZH2. Biochem Cell Biol. 2021;99(2):195–202. doi: 10.1139/bcb-2019-0497 32663416

[18] Song Z, Du J, Zhou L, et al. LncRNA AWPPH promotes proliferation and inhibits apoptosis of non‑small cell lung cancer cells by activating the Wnt/beta‑catenin signaling pathway. Mol Med Rep. 2019;19(5):4425–4432. 30942396 10.3892/mmr.2019.10089

[19] Chowdhury MAR, An J, Jeong S. The pleiotropic face of CREB family transcription factors. Mol Cells. 2023;46(7):399–413. doi: 10.14348/molcells.2023.2193 37013623 PMC10336275

[20] Cai Z, Aguilera F, Ramdas B, et al. Targeting BIM via a lncRNA MORRBID regulates the survival of preleukemic and leukemic cells. Cell Rep. 2020;31(12):107816. doi: 10.1016/j.celrep.2020.107816 32579941 PMC7371151

[21] Zhang S, Li C, Liu J, et al. Fusobacterium nucleatum promotes epithelial-mesenchymal transition through regulation of the lncRNA MIR4435-2HG/miR-296-5p/Akt2/SNAI1 signaling pathway. FEBS J. 2020;287(18):4032–4047. doi: 10.1111/febs.15233 31997506 PMC7540502

[22] Shen H, Sun B, Yang Y, et al. Mir4435-2hg regulates cancer cell behaviors in oral squamous cell carcinoma cell growth by upregulating TGF-beta1. Odontology. 2020;108(4):553–559. doi: 10.1007/s10266-020-00488-x 32016787

[23] Shi H, Cui W, Qin Y, et al. A glimpse into novel acylations and their emerging role in regulating cancer metastasis. Cell Mol Life Sci. 2024;81(1):76. doi: 10.1007/s00018-023-05104-z 38315203 PMC10844364

[24] Lou Y, Dong C, Jiang Q, et al. Protein succinylation mechanisms and potential targeted therapies in urinary disease. Cell Signal. 2025;131:111744. doi: 10.1016/j.cellsig.2025.111744 40090556

[25] Yang N, Li L, Shi XL, et al. Succinylation of SERCA2a at K352 promotes its ubiquitinoylation and degradation by proteasomes in sepsis-induced heart dysfunction. Circ Heart Fail. 2025;18(4):e012180. doi: 10.1161/CIRCHEARTFAILURE.124.012180 39996319

[26] Tan X, Wang S, Zhu L, et al. cAMP response element-binding protein promotes gliomagenesis by modulating the expression of oncogenic microRNA-23a. Proc Natl Acad Sci U S A. 2012;109(39):15805–15810. doi: 10.1073/pnas.1207787109 23019365 PMC3465427

[27] Mantamadiotis T, Papalexis N, Dworkin S. CREB signalling in neural stem/progenitor cells: recent developments and the implications for brain tumour biology. Bioessays. 2012;34(4):293–300. doi: 10.1002/bies.201100133 22331586

[28] Park JK, Park SH, So K, et al. ICAM-3 enhances the migratory and invasive potential of human non-small cell lung cancer cells by inducing MMP-2 and MMP-9 via AKT and CREB. Int J Oncol. 2010;36(1):181–192. 19956847

[29] Watson MJ, Berger PL, Banerjee K, et al. Aberrant CREB1 activation in prostate cancer disrupts normal prostate luminal cell differentiation. Oncogene. 2021;40(18):3260–3272. doi: 10.1038/s41388-021-01772-y 33846571 PMC10760404

[30] Wan X, Zhou M, Huang F, et al. Akt1-CREB stimulation of PDGFRalpha expression is pivotal for PTEN deficient tumor development. Cell Death Dis. 2021;12(2):172. doi: 10.1038/s41419-021-03433-0 33568640 PMC7876135

[31] Sapio L, Salzillo A, Ragone A, et al. Targeting CREB in cancer therapy: a key candidate or one of many? an update. Cancers (Basel). 2020;12(11). doi: 10.3390/cancers12113166 PMC7693618 33126560

