# Supplementary figures and images for: LncRNA MIR4435-2HG-mediated succinylation of USF1 promotes its protein stability and induces epithelial-mesenchymal transition in HNSCC
Source: Epigenetics. 2026 May 14;21(1):2672218. doi: 10.1080/15592294.2026.2672218 (PMC13178182; doi:10.1080/15592294.2026.2672218)

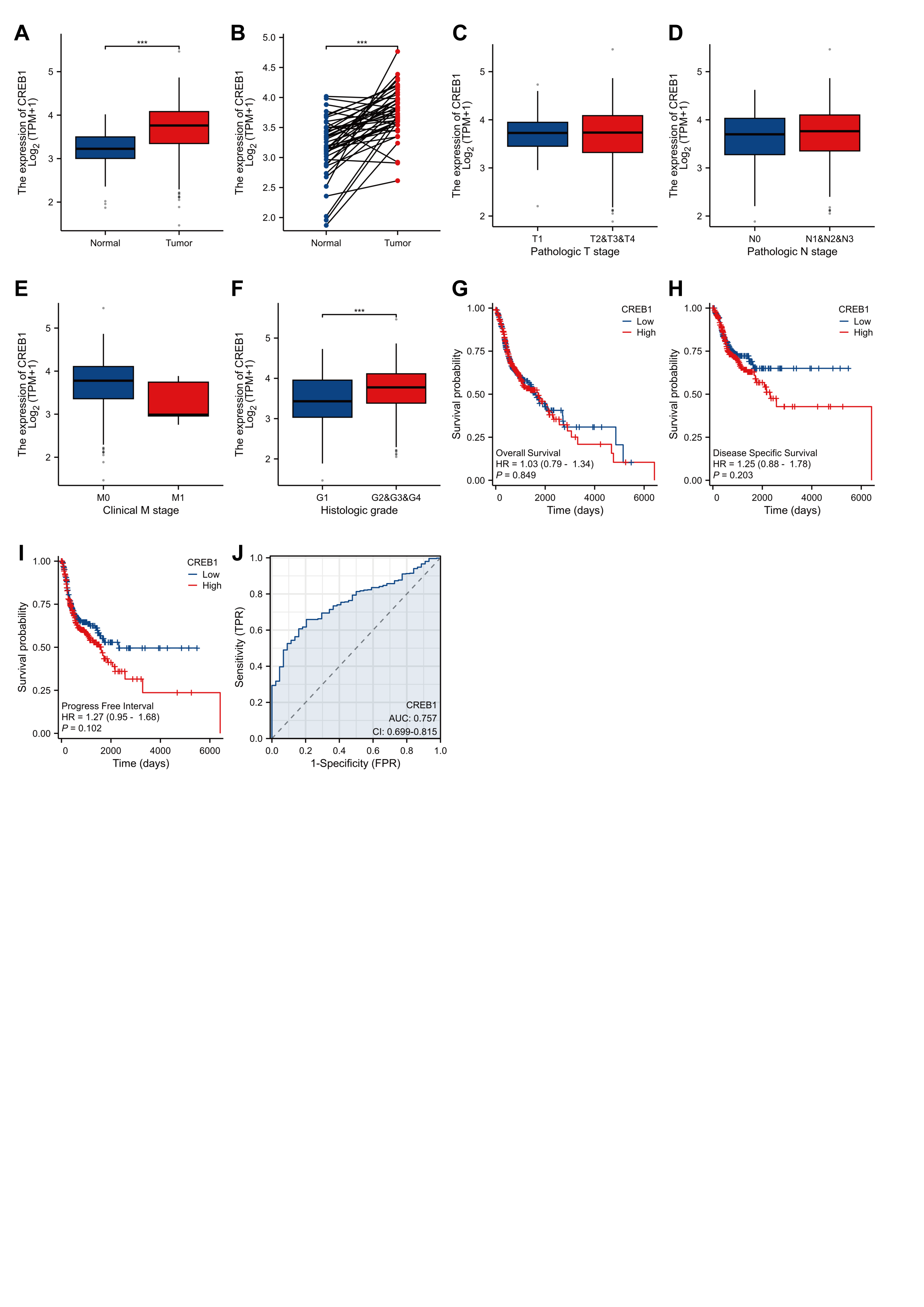

Supplement: Supplementary Figure 3.tif [file KEPI_A_2672218_SM6134.tif]

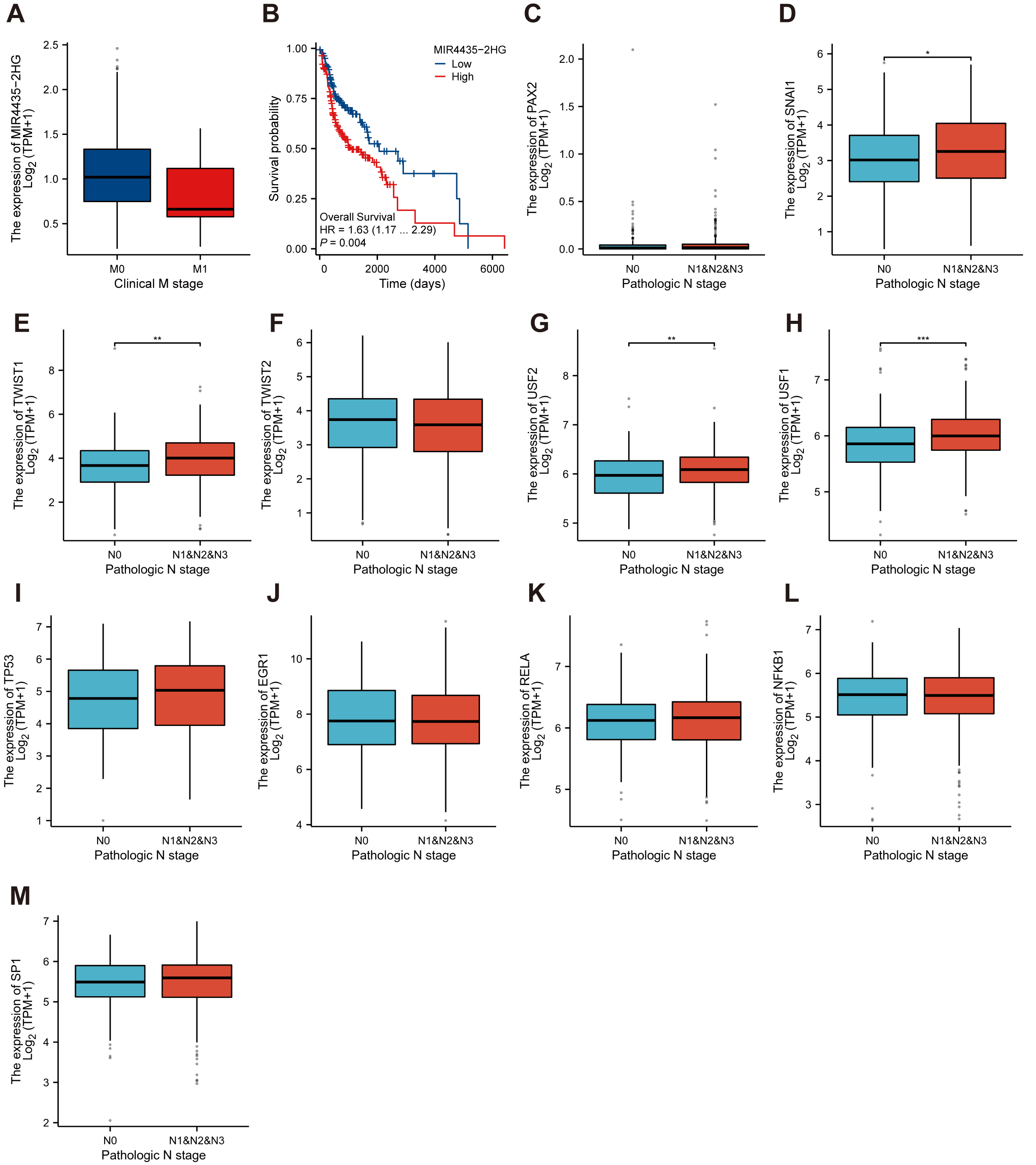

Supplement: Supplementary figure 1.tif [file KEPI_A_2672218_SM6133.tif]

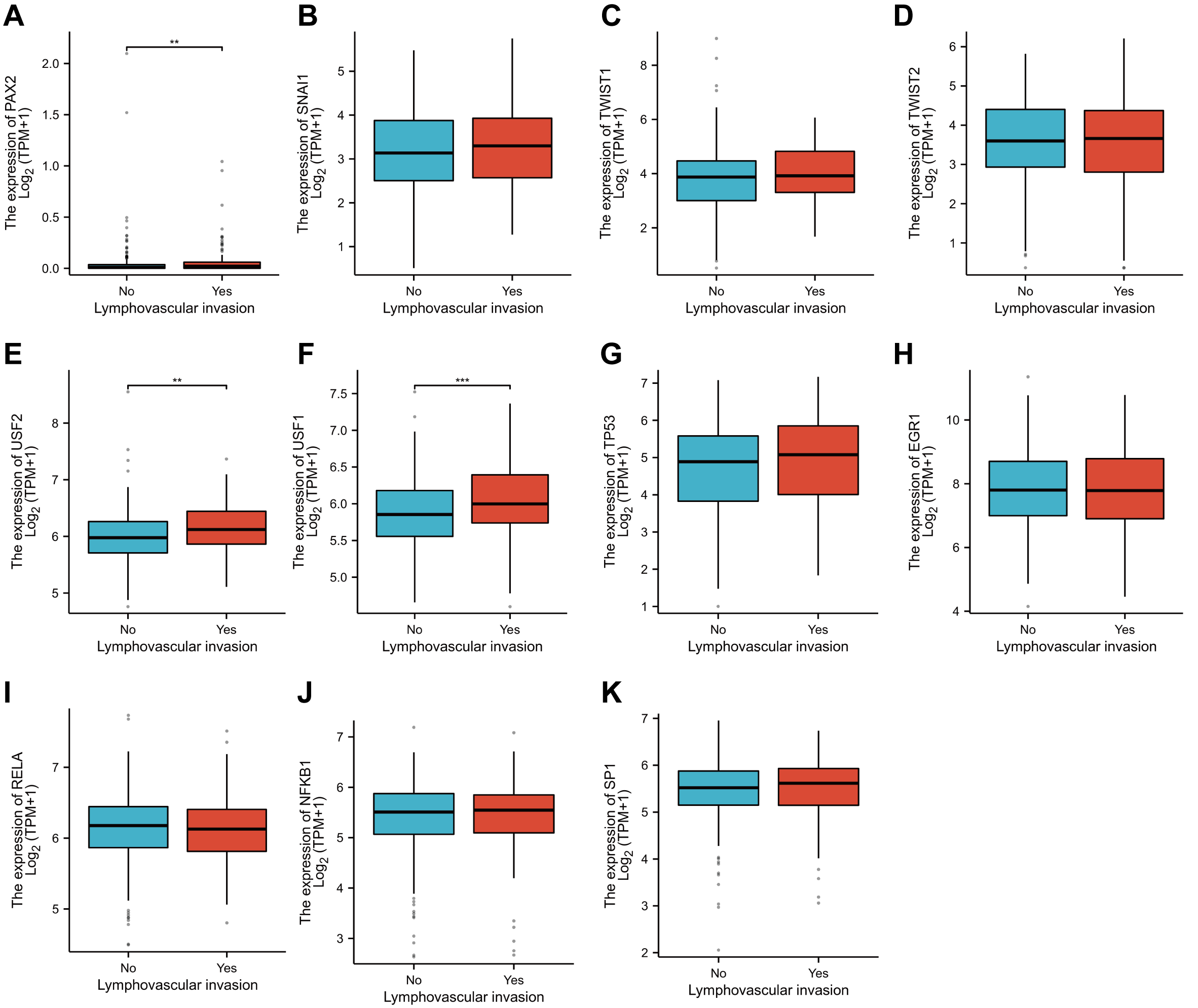

Supplement: Supplementary figure 2.tif [file KEPI_A_2672218_SM6130.tif]
