# Supplementary material for: LncRNA MIR4435-2HG-mediated succinylation of USF1 promotes its protein stability and induces epithelial-mesenchymal transition in HNSCC
Source: Epigenetics. 2026 May 14;21(1):2672218. doi: 10.1080/15592294.2026.2672218 (PMC13178182; doi:10.1080/15592294.2026.2672218)
Supplement: Supplementary Table 3.docx [file KEPI_A_2672218_SM6132.docx]

Supplementary Table 3. Cox regression analysis of CREB1 expression in the TCGA-HNSC cohort

| Characteristics | Univariate analysis | |  | Multivariate analysis | |
| --- | --- | --- | --- | --- | --- |
|  | Hazard ratio (95% CI) | P value |  | Hazard ratio (95% CI) | P value |
| Gender  (Male vs. Female) | 0.951 (0.687 - 1.316) | 0.762 |  |  |  |
| Age (<= 60 vs. > 60) | 1.088 (0.820 - 1.444) | 0.559 |  |  |  |
| Pathologic T stage  (T1 vs. T2&T3&T4) | 1.944 (1.397 - 2.704) | **< 0.001** |  | 2.251 (1.472 - 3.442) | **< 0.001** |
| Pathologic N stage  (N0 vs. N1&N2&N3) | 1.604 (1.148 - 2.241) | **0.006** |  | 1.405 (0.981 - 2.014) | 0.064 |
| Clinical M stage  (M0 vs. M1) | 2.842 (0.904 - 8.933) | 0.074 |  | 6.426 (1.945 - 21.230) | **0.002** |
| Clinical stage  (Stage I&II& III vs. Stage IV) | 1.260 (0.940 - 1.689) | 0.123 |  | 0.834 (0.570 - 1.221) | 0.350 |
| Histologic grade  (G1 vs. G2&G3&G4) | 1.363 (0.865 - 2.149) | 0.182 |  | 1.233 (0.688 - 2.208) | 0.482 |
| Radiation therapy  (No vs. Yes) | 0.843 (0.610 - 1.166) | 0.302 |  |  |  |
| CREB1  (Low vs. High) | 1.267 (0.954 - 1.683) | 0.102 |  | 1.428 (1.025 - 1.989) | **0.035** |
