# Supplementary material for: LncRNA MIR4435-2HG-mediated succinylation of USF1 promotes its protein stability and induces epithelial-mesenchymal transition in HNSCC
Source: Epigenetics. 2026 May 14;21(1):2672218. doi: 10.1080/15592294.2026.2672218 (PMC13178182; doi:10.1080/15592294.2026.2672218)
Supplement: Supplementary table 1.docx [file KEPI_A_2672218_SM6131.docx]

**Supplementary table 1. The primer sequences of RT-qPCR**

| Target genes | Upstream primer sequence | Downstream primer sequence |
| --- | --- | --- |
| MIR4435-2HG | 5'-GATCTTCACAGC  ACAGTTCCT-3' | 5'-GGTTGGAAAAGATGC  TGGTGA-3' |
| USF1 | 5'-GCTCTATGGAGAG  CACCAAGTC-3' | 5'-AGACAAGCGGTGGTTA  CTCTGC-3' |
| CREB1 | 5'-TTCTCCGGAACAC  AGATTTCA-3' | 5'-TGTCCATCAGTGGTCT  GTGC-3' |
| β-Actin | 5'-TTCCAGCCTTCC  TTCCTGG-3' | 5'-TTGCGCTCAGGAGGAG  CAAT-3' |
