# Supplementary material for: LncRNA MIR4435-2HG-mediated succinylation of USF1 promotes its protein stability and induces epithelial-mesenchymal transition in HNSCC
Source: Epigenetics. 2026 May 14;21(1):2672218. doi: 10.1080/15592294.2026.2672218 (PMC13178182; doi:10.1080/15592294.2026.2672218)
Supplement: Supplementary table 2.docx [file KEPI_A_2672218_SM6129.docx]

SupplementaryTable 2 . Univariate and multivariate logistic regression analyses in HNSCC

| Characteristics | Univariate analysis | |  | Multivariate analysis | |
| --- | --- | --- | --- | --- | --- |
|  | Hazard ratio (95% CI) | P value |  | Hazard ratio (95% CI) | P value |
| Gender  (Male vs. Female) | 0.760  (0.571-1.012) | 0.061 |  | 0.866  (0.594 - 1.265) | 0.457 |
| Age (<= 60 vs. > 60) | 1.262  (0.964 - 1.653) | 0.090 |  | 1.219  (0.854 - 1.740) | 0.276 |
| Pathologic T stage  (T1 vs. T2&T3&T4) | 2.006  (1.091 - 3.688) | **0.025** |  | 2.222  (1.060 - 4.656) | **0.034** |
| Pathologic N stage  (N0 vs. N1&N2&N3) | 1.853  (1.334 - 2.572) | **< 0.001** |  | 2.102  (1.419 - 3.113) | **< 0.001** |
| Clinical M stage  (M0 vs. M1) | 4.769  (1.757 -12.947) | **0.002** |  | 4.612  (1.084 19.628) | **0.039** |
| Clinical stage  (Stage I&II& III vs. Stage IV) | 1.165  (0.888 - 1.530) | 0.270 |  |  |  |
| Histologic grade  (G1 vs. G2&G3&G4) | 1.582  (1.018 - 2.458) | **0.041** |  | 1.285  (0.709 - 2.331) | 0.408 |
| Radiation therapy  (No vs. Yes) | 0.617  (0.455 - 0.837) | **0.002** |  | 0.449  (0.306 - 0.660) | **< 0.001** |
| MIR4435-2HG  (Low vs. High) | 1.431  (1.093 - 1.874) | **0.009** |  | 1.530  (1.068 - 2.190) | **0.020** |
